# The Clinical Relevance of Pre-Formed Anti-HLA and Anti-MICA Antibodies after Cord Blood Transplantation in Children

**DOI:** 10.1371/journal.pone.0072141

**Published:** 2013-08-19

**Authors:** Marc Ansari, Chakradhara Rao S. Uppugunduri, Sylvie Ferrari-Lacraz, Henrique Bittencourt, Fabienne Gumy-Pause, Yves Chalandon, Jean-Marie Tiercy, Tal Schechter, Adam Gassas, John D. Doyle, Lee Dupuis, Michel Duval, Maja Krajinovic, Jean Villard

**Affiliations:** 1 Onco-Hematology Unit, Department of Pediatrics, Geneva University Hospital, Geneva, Switzerland; 2 CANSEARCH Research Center, Geneva Medical University, Geneva, Switzerland; 3 Transplantation Immunology Unit and Swiss National Laboratory for Histocompatibility, Division of Immunology and Allergy, Division of Laboratory Medicine, Geneva University Hospital and Medical School, Switzerland; 4 Oncology and Hematology Unit, Department of Pediatrics, CHU Ste Justine, Montreal, Canada; 5 Canada Department of Pharmacy, Division of Hematology/Oncology, The Hospital for Sick Children, University of Toronto, Toronto, Canada; 6 Charles-Bruneau Cancer Center, CHU Sainte-Justine Research Center, Montreal, QC, Canada; 7 Clinical Pharmacology Unit, CHU Sainte-Justine, Montreal, QC, Canada; Albert Einstein Institute for Research and Education, Brazil

## Abstract

Preformed anti-HLA antibodies (AHA) are known to be associated with delayed engraftment and reduced overall survival after adult hematopoietic stem cell transplantation. However, limited data is available in pediatric patients. In this study, we explored the role of AHA on clinical outcomes in 70 pediatric patients who received a single unit of HLA mismatch cord blood for hematologic malignancies, immunodeficiencies or metabolic diseases. The presence of AHA was detected in 44% (31/70) of the patients. Preformed class I AHA was associated with an increased occurrence of grade 1–4 acute graft-versus host disease (p<0.05). The presence of anti- major-histocompatibility-complex class I–related chain A antigens (MICA) antibodies was significantly associated with a reduced platelet recovery after transplantation (p<0.05). AHA of class II with the strength of antibody titer measured as the mean fluorescence intensity above 2000 was associated with reduced event-free survival (p<0.05). A reduction of high titer of AHA and anti-MICA antibodies might have to be considered before cord blood transplantation in pediatric patients for better outcomes.

## Introduction

The presence of preformed anti-HLA antibodies (AHA) is a risk factor for antibody mediated rejection and is associated with reduced clinical outcomes in solid organ transplantation, especially in kidney transplantation [1]–[6]. Therefore, detection and determination of specific anti-HLA are a part of the preparatory tests performed by the laboratory before kidney transplantation [1].

Investigating the role of AHA in determining clinical outcomes of hematopoietic stem cell transplantation (HSCT) has recently gained interest. Although the objective of HSCT is to select a complete HLA matched donor, currently more transplants are being performed with partially matched donors with the availability of cell sources from umbilical cord blood, unrelated donors from the worldwide registry and haploidentical donors. In adults, the presence of AHA directed against HLA-mismatched donors has been associated with graft failure, delayed neutrophil, platelet recoveries, and graft versus host disease (GVHD), leading to reduced overall survival (OS) [7]–[16]. In children, cord blood unit transplantations became more frequent and are preferred in situations of HLA mismatch. To the best of our knowledge clinical relevance of preformed AHA in pediatric transplantations with cord blood as the only source has not been reported until now. Similar to the reports from adult HSCT patients available in the literature, the presence of preformed AHA, arising mainly due to blood transfusions, could also be deleterious in clinical outcomes of HSCT in children. In addition to AHA, preformed antibodies against major-histocompatibility-complex class I–related chain A antigens (anti-MICA antibodies) might also be detrimental in HSCT outcomes.

In this explorative study, we had the opportunity to analyze the presence of AHA and anti-MICA antibodies in a cohort of 70 children receiving single cord blood transplantation. Using the Luminex technology, we investigated the presence of AHA of class I, II, and anti-MICA antibodies prior to HSCT, and correlated them with clinical outcomes.

## Materials and Methods

### Umbilical Cord Blood Transplantation

This study comprise 70 children, 53 of them underwent allogeneic HSCT at CHU Sainte-Justine, Montreal between May 2000 and August 2010 and 17 children underwent allogeneic HSCT at the Hospital for Sick Children, Toronto, between July 2008 and October 2009.

The patients received a busulfan (Bu) based conditioning regimen either myeloablative (94.3%, n = 66) or non-myeloablative (5.7%, n = 6) (Table 1). Intravenous (iv) Bu (Busulfex®, Otsuka Pharmaceuticals) first dose was based on age of the patient and a pharmacokinetics guided dose adjustment was subsequently performed [17], [18]. The majority of the patients received iv cyclophosphamide (200 mg/kg total dose; table 1) following Bu administration. GVHD prophylaxis was provided with cyclosporine to all the patients, with the addition of either, methotrexate, mycophenolic acid, steroids and mycophenolic acid combinations to 92.5% (49), 5.6% (3), 1.2% (1), and 1.2% (1) of the patients, respectively (data available for 53 patients). Sixty-four (94.3%) patients received anti-thymocyte globulin. Granulocyte colony-stimulating factor (G-CSF) was given to all CHU Sainte-Justine patients after each cord blood infusion but not to patients from the SickKids Hospital. Prophylaxes against fungal, viral, *Pneumocystis jiroveci* infections were administered as per institutional standards (fluconazole, acyclovir and trimethoprim/sulfamethoxazole) and ursodeoxycholic acid was given as a veno-occlusive disease (VOD) prophylaxis only to CHU St-Justine patients. Seizure prophylaxis was provided with phenytoin (26.9%), midazolam (19.4%) or lorazepam (53.7%). All patients received a single umbilical cord blood (UCB) unit. The HLA matching between the UCB unit and the recipient was at least 4 out of 6 for the majority of cases (>97%) at an antigen level of HLA-A, B and allelic level of DRB1 (see table 1).

**Table 1:** Demographic characteristics of whole cohort.

Demographic characteristics		Whole cohort
**Number of patients** n (%)		70 (100)
**Age (Years)**	Mean (median)range	6.5 (5.0) 0.1–19.9
**Body weight (Kg)**	Mean (median)range	27.0 (20.8) 4.3–95.6
**Gender** n (%)	Male	41(58.6)
	Females	29 (41.4)
**Diagnosis** n (%)	AML	26 (37.1)
	MDS	12 (17.1)
	Immunodeficiency	11 (15.7)
	ALL	9 (12.9)
	Metabolic disease	5 (7.1)
	Hemophagocytic syndrome	5 (7.1)
	Hemoglobinopathies	1 (1.4)
	Neuroblastoma	1 (1.4)
**Conditioning regimen** n (%)	Bu+Cy	53 (75.7)
	Bu+Cy+Mel	3 (4.3)
	Bu+Cy+VP16	7 (10)
	Bu+Flu	4 (5.7)
	Bu+Mel	3 (4.3)
**Disease status in malignancies** n (%)	First acute phase of the disease	1(2.7)
	First CR	18(50.0)
	second CR	12(33.3)
	third or more CR	2(5.5)
	First Relapse	3(8.3)
**HLA Matching** n (%)	3/6	2 (2.9)
	4/6	20 (28.6)
	5/6	28 (40)
	6/6	16 (22.9)
	7/8	1 (1.4)
	8/8	2 (2.9)
	7/10	1 (1.4)
**HLA match compatibility** n (%)	MRD	2(2.8)
	MUD	16 (22.9 )
	MMU	52 (74.3)
**Days after transplant**	Mean (median)	1064.4 (815.0)
**Nucleated cells injected (10^8^/kg)**	Mean (median)range	2.1 (1.4) 0.16–18.0
**CD34^+^ cells injected (10^8^/kg) n = 64**	Mean (median)range	0.02 (0.004) 0.0005–0.14

Abbreviations: ALL: acute lymphoblastic leukemia; AML: acute myeloid leukemia; ATG: anti-thymocyte globulin; Bu: busulfan; Cy: cyclophosphamide; CR: Complete remission; Flu: fludarabine; MDS: myelodisplastic syndrome; Mel: melphalan; MMU: mismatch unrelated; MRD: matched related donor; MTX: methotrexate; MUD: matched unrelated donor; VP16: etoposide.

GVHD grading was based on the 1994 Consensus Conference on Acute GVHD [19]. Neutrophil recovery was defined as the first of 3 consecutive days with an absolute neutrophil count of 0.5×10^9^/L or higher. Platelet recovery was defined as the first of 7 consecutive days with platelet counts higher than 50×10^9^/L without transfusion. Primary graft failure was defined by persistent pancytopenia with no evidence of hematologic recovery of donor cells beyond 28 days after transplantation and secondary graft failure by a rapid decrease in neutrophil count after successful engraftment. Event-free survival (EFS) was calculated from time of transplant until death, relapse or graft failure, whatever occurred first. Overall survival (OS) was the time between transplantation and death due to any cause.

### Anti-HLA Antibody Assays

Plasma samples collected prior to the conditioning regimen were tested with the screening assay LAB Screen® Mixed (One Lambda Inc., Canoga Park, CA) according to manufacturer’s instructions. Test interpretation was performed using HLA Visual® software (One Lambda) on the LABScan100™ flow cytometer (Luminex Inc., Austin, TX) with a positive cut off of 3.0. Anti-MICA antibodies were also determined by the same technology and are included in the LABScreen® Mixed kit.

To identify the specificity of AHA, high definition LAB Screen® Single Antigen (One Lambda) class I or class II assays were performed with plasma of recipients who tested positive for the LAB Screen® Mixed assay. Results were interpreted using the LABS can™ 100 software (One Lambda) on the LABScan100™ flow cytometer (Luminex). A positive result was defined when a mean fluorescence intensity (MFI) was above 1000 and calculated as follows: (fluorescence of beads coated with HLA and incubated with patient plasma) – (fluorescence of beads without HLA and incubated with patient plasma) – (fluorescence of beads with and without HLA and incubated with negative control plasma).

### Statistical Analysis

The distribution of demographic characteristics between the groups with and without antibodies was compared using *Chi*-square test for categorical variables and non-parametric tests (Mann-Whitney and Kruskal –Wallis rank tests) for continuous variables. Cumulative incidences of neutrophil and platelet recoveries were estimated using the cumulative incidence (CI) function, with death as a competitive event. Cumulative incidences in the presence of the competing risk were compared between the groups using the Gray’s test in a univariate analysis, and Fine and Gray multivariate regression model [20], [21]. Probabilities of survival i.e. OS and EFS were estimated using Kaplan-Meier curves and log-rank test by comparing the differences between groups in a univariate analysis. Hazards ratios (HR) with 95% confidence intervals (CI) were estimated using univariate cox-regression and impact of antibody status was assessed in multivariate cox-proportional hazards regression analysis. In multivariate analyses co-variates were included as categorical models such as diagnosis (malignant, non-malignant), number of nucleated cells infused (above and below the median value), number of CD34^+^ cells infused (above and below the median value), G-CSF receiving status (received versus not received) and conditioning regimen (myeloablative, non-myeloablative). For aGVHD (grade 1–4) cumulative incidence, in addition to the above co-variates, GVHD prophylaxis (cyclosporine and steroids versus other prophylaxis), serotherapy (received versus not received) and HLA matching status (100% match versus less than 100% match) were included in the multivariate analysis. Two sided p-values are presented and a probability less than 0.05 was considered as significant. Analyses were performed using the IBM® SPSS® statistical package (version 19, SPSS Inc, NY) and R statistical software (http://r-project.org).

## Results

### Patient Characteristics

Seventy patients from two study centers were included in the analysis. The patient demographic characteristics are given in table 1. Most of the patients (47/70) were transplanted for hematopoietic malignancies, 1 patient for a neuroblastoma and the others for immunodeficiencies (11/70), metabolic diseases (5), genetic related hemophagocytic syndrome (5) or hemoglobinopathy (1). Among the leukemic patients (36), 25.7% (18), 17.1% (12) and 2.9% (2) were in first; second and third complete remission, respectively. Three patients were in first relapse and 1 patient was in acute phase of the disease. HLA mismatch profiles are depicted in table 1. The mean (median) number of infused total nucleated cells was 2.1×10^8^/kg (1.4×10^8^/kg) and for 64 patients where there was data on the number of CD34^+^ cells infused the mean (median) calculated to 0.02×10^8^ cells/kg (0.004×10^8^/kg).

The incidences of overall OS, EFS, neutrophil, and platelet recoveries were 62.9%, 45.7%, 84.3%, and 65.7%, respectively. Median time to neutrophil engraftment was 20 days (mean = 22.9 days), and median time to platelet recovery was 58.5 days (mean  = 60.2 days). VOD, hemorrhagic cystitis, acute GVHD (grade 1–4) and lung toxicity incidences were 7.1%, 28.6%, 24.3%, and 7.1%, respectively. Eight patients had primary and three had secondary graft failures. The median follow up of the cohort was 26.8 months (mean  = 35.2 months). A total of 26 patients died with 3 patients before neutrophil engraftment, and 6 patients before platelet recovery.

### Preformed anti-HLA and anti-MICA Antibodies

To validate the assay using plasma (instead of serum, according to the classical assay from the manufacturer), 10 serum samples and 10 plasma samples were tested from immunized patients for the presence of AHA and anti-MICA antibodies. In line with previously reported data [22], our results confirmed an excellent concordance between the two types of samples (data not shown).

AHA were detected in 44.3% of the patients among which 58.1% were positive for antibodies against either HLA class I, or class II AHA and 41.9% were positive for both class I and II (Table 2). Donor specific antibodies (DSA) were detected in 12 individuals (4 for class I, 5 for class II and 3 for both classes). Anti-MICA antibodies were detected in 11 patients; in 8 patients, anti-MICA antibodies status was undetermined (borderline to the limit of detection). We did not observe any statistical differences with regards to the presence of pre-formed AHA or anti-MICA antibodies and demographics, or any clinical parameter of the patients before transplantation like age, gender, conditioning regimen or type of disease (data not shown), except that patients detected for AHA for class I were infused with a lower number of CD34^+^ cells compared to the remaining patients (p<0.05).

**Table 2:** Patient demographic characteristics distribution among groups based on anti-HLA antibodies, DSA and MICA detection status.

Demographic characteristics		Anti-HLA antibodies detection status							
		Not detected	Class IAHA	Class IIAHA	Both	DSA negative	DSA positive	MICA negative	MICA positive
**Number of patients**		39	22	22	13	58	12	51	11
**Age** **in years, Mean (median)**		7.4 (5.2)	5.2 (3.2)	5.7 (5.9)	5.9 (6.2)	6.5 (4.6)	6.4 (5.9)	6.4 (5.0)	6.3 (4.4)
**Body weight, Kg, Mean (median)**		29.8 (23.2)	23.2 (15.8)	25.1 (24.1)	25.8 (26.9)	26.9 (18.8)	27.3 (25.1)	26.7 (21.5)	28.3 (20.1)
**Gender (**n)	MaleFemales	2316	1111	1210	58	3622	57	3021	83
**Diagnosis(**n)	AML	15	9	7	5	21	5	16	7
	MDS	7	1	5	1	10	2	11	1
	Immunodeficiency	4	6	5	4	8	3	6	2
	ALL	7	0	2	0	7	2	9	0
	Metabolic disease	2	3	2	2	5	0	3	1
	Hemophagocytic syndrome	2	3	1	1	5	0	5	0
	Hemoglobinopathhies	1	0	0	0	1	0	0	0
	Neuroblastoma	1	0	0	0	1	0	1	0
**Nucleated cells injected X10^8^/kg)** **Mean (median) range**		2.1 (1.4)0.16–18.0	1.8 (1.1)0.29–9.0	2.8 (1.4)0.29–14.80	2.5 (1.3)0.29–9.0	1.9 (1.4)0.16–18.0	3.2 (1.3)0.29–14.80	2.3 (1.4)0.16–18.0	1.3 (0.9)0.33–3.15
**CD34^+^ cells injected (X10^8^/kg)** **N = 64** **Mean (median) range**		0.02 (0.005)0.0004–0.13	0.005 (0.002)0.0005–0.046	0.02 (0.004)0.0007–0.14	0.007 (0.002)0.0007–0.05	0.01(0.004)0.0004–0.14	0.02 (0.002)0.0005–0.11	0.02 (0.004)0.0007–0.14	0.007 (0.003)0.0004–0.041

Abbreviations: AHA : anti-HLA antibodies;ALL: acute lymphoblastic leukemia; AML: acute myeloid leukemia; ATG:anti-thymocyte globulin; Bu: busulfan; Cy: cyclophosphamide; DSA : Donor-specific anti-HLA antibody; Flu: fludarabine; MDS: myelodisplastic syndrome; Mel: melphalan; MICA : major-histocompatibility-complex class I–related chain A antigens MM: mismatch; MRD: matched related donor; MTX: methotrexate;MUD: matched unrelated donor; VP16:etoposide. AHA of either classes were detected in 31 patients. Class I AHA and class 2 AHA alone were detected in 9 patients, and both in 13 patients, thus 22 (9+13) patients were positive for class I AHA and class II AHA. MICA antibody data was available for only 62 patients out of 70. Data for infused number of CD34^+^ cells was available for only 64 patients out of 70.

### Effect of Anti-HLA Antibodies and anti-MICA Antibodies on Hematologic Recovery and other Clinical Outcomes

**Figure 1:**
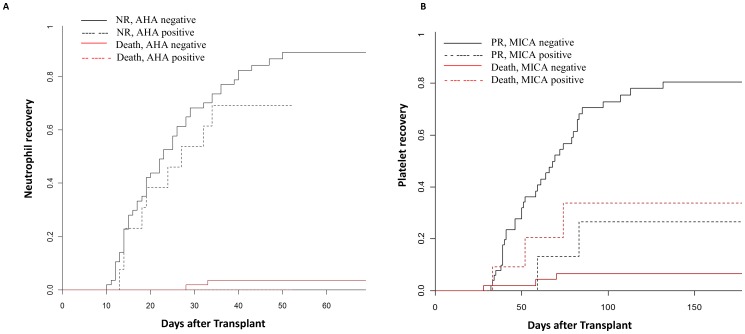
Cumulative incidence of neutrophil and platelets recovery. A) 69.3% (95%CI: 58.49–80.11%) of patients positive for both class I and II anti HLA antibodies (AHA) had neutrophil recovery (NR) compared to 89.3% of negative patients (95% CI: 82.06–96.54%). NR did not differed in both groups, Gray’s test p-value = 0.21, hazards ratio is 1.6 (0.8–3.3). No patients detected for both class AHA were dead before NR. B) Patient’s positive for anti major-histocompatibility-complex class I–related chain A antigen (MICA) antibodies had lower cumulative incidences of platelet recovery (PR, 26.5% (95% CI: 15.51–37.49%) compared to negative patients (80.5%; 95% CI: 70.64–90.36%).Total number of patients included in analysis was 62; death before PR was used as competing event, and for 8 patients we do not have conclusive MICA status. Gray’s test p value = 0.04, hazards ratio is 4.3 (1.02–17.8).

Patients detected for AHA of both classes had a trend of lower incidence of neutrophil recovery (69.3%, 9/13 engrafted, 0/13 dead before recovery) compared to patients negative for both classes of AHA (89.3%, 50/57 engrafted, 3/57 dead before recovery, p = 0.21; HR:1.6 (0.8–3.3), Figure 1A). In a multivariate regression analysis including AHA status, CD34^+^cell number infused, G-CSF receiving status, age and gender, only CD34^+^ cell number above the median was associated with low neutrophil recovery (p = 0.023). AHA status showed a trend of association (p = 0.15). No significant differences in neutrophil engraftment was observed between patients who received G-CSF (84% engraftment) compared to those not received (82% engraftment). The presence of preformed anti-MICA antibodies was significantly associated with a lower incidence of platelet recovery (26.5%) compared to those who were negative (80.5%) for anti-MICA (p = 0.04; HR: 4.2 (1.02–17.08), Figure 1B). Patients diagnosed for malignant condition also had lower incidences of platelet recovery (63.6%) compared to those with non-malignant disease (90%) (p<0.0001, HR: 3.5 (1.9–6.6)). In a multivariate regression analysis including MICA status, AHA status, nucleated cell number and diagnosis, MICA positive status showed a trend of association with lower platelet recovery (p = 0.06), whereas diagnosis was significantly associated with lower platelet recovery (p = 0.003). Dead patients were considered as a competing risk in univariate and multivariate analysis for neutrophil and platelet recoveries, when they died before recovery.

### Influence of anti-HLA Antibodies and their Titers on aGVHD, Event Free Survival or Overall Survival

**Figure 2:**
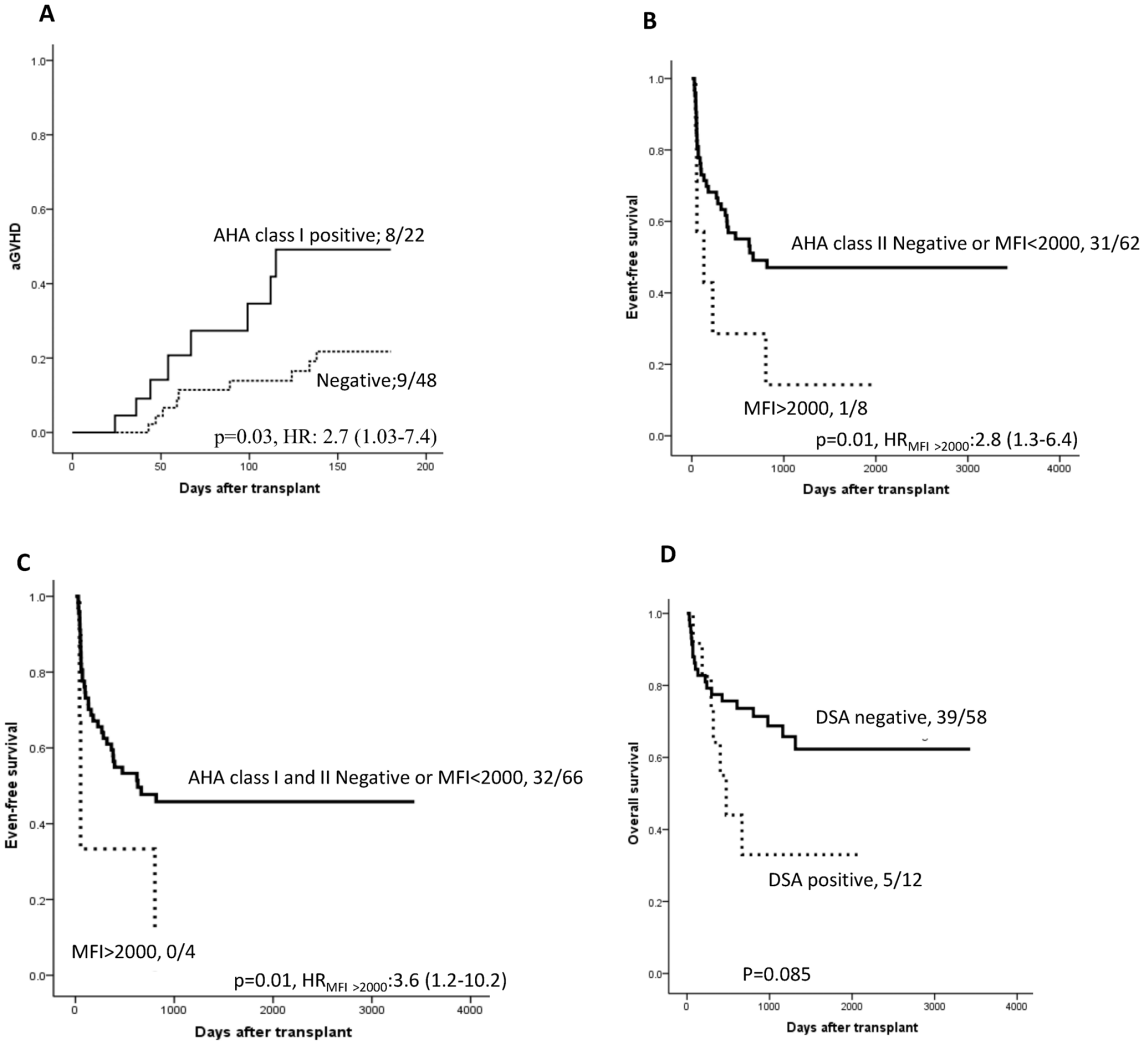
Cumulative incidence of acute GVHD (grade 1–4), event-free survival and overal survival. The number of patients with aGVHD occurrence or with no events or survived/total number of patients in each group, hazards ratios with 95% confidence interval is presented on the plot. A) Patients positive for class I AHA had higher incidence of aGVHD (36.4%) compared to those negative for class I AHA (18.7%). B) Patients positive for class II AHA with mean florescence intensity (MFI) above 2000 had lower event free survival (12.5%) compared to those negative or positive with MFI less than 2000 (50%). C) Patients detected for both class I and II AHA had lower EFS (0%) compared to those negative or positive with MFI below 2000 (48.5%). D) Patients with DSA against the HLA mismatch of the cord blood had a trend of lower overall survival (41.6%) compared to patients without DSA (67.3%).

Patients detected for class I AHA had a higher incidence of acute GVHD compared to the remaining patients (36.4% Vs 18.7%; p = 0.03, HR: 2.7 (1.03–7.4); Figure 2A). In univariate analysis, CD34^+^ cell number above the median, myeloablative conditioning regimen and serotherapy showed trends of association with higher incidence of aGVHD. No aGVHD was seen in patients receiving non-myeloablative conditioning regimens. Interestingly, HLA match showed (categorical) no association with aGVHD. A higher incidence of aGVHD was seen in patients who did not received serotherapy (50% Vs 23%, p = 0.06). In a multivariate analysis including GVHD prophylaxis, serotherapy, CD34^+^ cell number, conditioning regimen and disease, class 1 AHA remained independently associated with the occurrence of aGVHD1-4 (p = 0.024).

The presence of preformed class II AHA was associated with reduced EFS. The presence of class II AHA with MFI >2000 was associated with a lower EFS compared to those negative for class II AHA or positive but with MFI <2000 (12.5% vs. 50% p = 0.01, HR:2.8 (1.3–6.4); Figure 2 B). Lower EFS (HR: 3.6 (1.2–10.2)) was seen in individuals detected for both class I and II AHA at MFI>2000 compared to the remaining patients (0% vs. 49% p = 0.01, Figure 2 C).

A trend for lower OS (25% vs. 50%; p = 0.085; Figure 2D) was observed in patients positive for DSA (n = 12), compared to patients with no DSA (n = 58). When analyzed for impact of AHA and DSA status on OS, we observed 66.7% OS in patients with no AHA detected; 68.4% OS in patients with AHA detected but not donor specific whereas patients detected with DSA had 41.7% OS (log-rank p value = 0.2).

Among other factors known to influence OS and EFS patients diagnosed with a malignant disease demonstrated a lower EFS (p = 0.007), whereas nucleated cell number and CD34+ cell number showed only a trend of association in a univariate analysis (p = 0.2–0.4). In a multivatriate analysis only diagnosis showed a significant association with EFS, whereas class II AHA with MFI >2000 showed only a trend.

## Discussion

In this retrospective analysis, we have demonstrated the interest of detecting preformed AHA and anti-MICA antibodies in a pediatric cohort receiving cord blood unit transplantation. Our data shows the importance of preformed anti-MICA antibodies, which appears to be associated with lower cumulative incidences and prolonged times to platelet recovery. The presence of class I AHA showed an associated with acute GVHD incidence. Preformed AHA of class II was also associated with reduced EFS when MFI is above 2000. These observations highlight the importance of AHA in determining EFS in cord blood transplants which has been shown to result in similar disease free survival as that of bone marrow transplant in children [23]. The data presented here also highlights that the delay in engraftment with cord blood transplant will be further augmented by the presence of AHA.

The analysis included all patients with both myeloablative and non-myeloablative (n = 4) conditioning regimens. The statistical significance was not altered when analyzed with patients receiving myeloablative-conditioning regimen only.

The major limitation of our study is that the sample size is small, which reduces statistical power. The power of the study was 50 to 60%, calculated for the observed incidences of neutrophil recovery and EFS in the study sample at a probability (alpha) of 0.05. Due to the small sample size, we were only able to pick up trends in a few of the associations described. These associations and trends observed in this study must be confirmed in a larger cohort to establish a real association. However, we believe that our results are of interest and specific to a pediatric population, transplanted with a single cord blood unit, since this is the largest cohort ever investigated for this topic in the best of our knowledge.

The level of immunization in our pediatric cohort was quite high (44%), compared to existing data on adults [11]. The cohort is mostly comprised of patients with malignant condition, especially leukemia which usually necessitate transfusions. This consequently might result in alloimmunization and adverse outcomes. Unfortunately, we do not have the transfusion data from these patients. Since malignant disease status itself was independently associated with less survival, we did not include stage of leukemia’s in the multivariate analysis owing to a low number of patients. However, it would be interesting to prospectively evaluate the degree of alloimmunization in relation to the disease status and the level of transfusions within a large homogeneous cohort before transplantation.

The presence of anti-MICA antibodies was associated with reduced platelet recovery. MICA encodes the highly polymorphic MHC (HLA) class I chain-related gene A. Although unlike classical MHC class I molecules MICA does not seem to be associate with beta-2-microglobulin [24] with functions related to innate immunity [25]–[27]. MICA antigens are expressed on the surface of endothelial cells, dendritic cells, fibroblasts, epithelial cells, activated CD4^+^ T, CD8^+^ T cells and as well as in many tumors. MICA functions as a stress-induced antigen that is broadly recognized by Natural killer (NK) cells, NKT cells, and T cells. MICA acts as a ligand for the NK cell activating receptor NKG2D [25]–[27]. Solid organ transplantation, pregnancy, blood transfusion [28], and other mechanism like cross reactivity with substances from the environment [29] could explain the development of anti- MICA antibodies that can be detected by different technologies like ELISA or Luminex [30]. In renal transplantation, the presence of anti-MICA antibodies is associated with renal allograft rejection and reduced graft survival [31], [32]. In HSCT, the data for the role of anti-MICA antibodies is limited [33], [34], but in adult, Parmar *et al*., demonstrated an association between the presence of anti-MICA antibodies and acute GVHD [33]. In our study, the influence of anti-MICA antibodies on platelet recovery was independent of the presence of AHA of class I and/or class II. Typing the platelets of donors for MICA antigens could not be performed because donor DNA was not available, hence formal proof of donor specificity could not be obtained. To the best of our knowledge, there is no data on the expression of MICA by platelets available in literature.

We observed a higher incidence of acute GVHD (grade 1–4) in patients positive for class I AHA. Overall there is a trend of higher incidence of acute GVHD in patients positive for AHA irrespective of their class and titers. Though the incidences and severity of acute GVHD are lower in cord blood transplant compared to bone marrow transplant, our data demonstrates that this could be further lowered in patients negative for class I AHA [23]. The distribution of prophylaxis for GVHD was similar in groups positive and negative for AHA with most of them receiving cyclosporine and steroid based prophylaxis (data available for 53 patients only). Association of AHA with GVHD has not been found in previous studies conducted in adults or mixed cohorts [13], [15]. AHA can participate in the inflammation of the tissue of the recipients by a direct binding or by an indirect mechanism and play a role in the GVHD process. It is interesting to note that patients detected for AHA of class I received a significantly lower number of CD34^+^ cells compared to those who were negative for class I AHA. We did not find an independent association of AHA class I status and engraftment but CD34^+^ cell numbers below the median was independently associated with a delayed engraftment, irrespective of AHA status. Moreover, AHA class I status was independently associated with aGVHD, but not CD34^+^ cell number (showed only a trend). Engraftment was delayed in patients detected for both classes of AHA compared to those who are positive for either one class or negative for AHA, suggesting an important role of class II AHA.

Our data indicates that the presence of AHA with high MFI are more deleterious with regards to EFS in cord blood transplant patients. This is important because this suggests that a strategy could be implemented to keeping or lowering the level of AHA below MFI  = 2000, using the antibody titer. The clinical utility of antibodies titer defined by MFI is a subject of debate in solid organ transplantation [1], [35], [36]. Although the test is only certified by the regulatory agencies for qualitative results, there is increasing evidence in the literature that MFI allows stratification for the risk of clinical event [1], [15], [36].

Our study suggests that the reduced EFS was mainly linked to AHA of class II. In contrast to MHC class I, MHC class II is not expressed constitutively on any cell type. Only specialized cells express MHC class II, like antigen presenting cells, B cells or thymic epithelial cells. However, MHC class II can be induced by pro-inflammatory cytokines like γ-interferon or TNFά. The expression of MHC class II on any tissue reflects a state of inflammation. Therefore we hypothesize that in addition to the specific and direct effect of donor specific antibodies, global inflammation related to conditioning regimen before transplantation and any event after transplantation (like infection, GVHD, mucositis, hemorrhagic cystitis) can induce the expression of MHC class II. This indirectly reflects a state of inflammation associated with the occurrence of clinical events such as rejection, relapse and infections and finally reduced EFS.

Patients either detected for AHA which are not donor specific or not detected for AHA had similar OS, whereas patients detected for AHA which are donor specific had lower OS, but this observed difference is not statistically significant. As mentioned above, this could be explained by the limited number of patients in the cohort, but is also due to the fact that some of the DSA have a low antibody titer (MFI<2'000). Of note, DSA against HLA-Cw, HLA-DQ and HLA-DP were not taken into consideration because HLA typing for these loci were not available for every patient. This observation is of importance, because it is likely that our ability to assess DSA is not completely accurate and some antibodies deemed DSA-negative in fact might have had reactivity against the donor. This is also consistent with the observation of multiple epitope sharing among HLA-II molecules [37].

In conclusion, our data strongly suggests that in children the presence of preformed high titer of AHA and anti-MICA antibodies are associated with reduced hematologic recovery and reduced event-free survival. The presence of class I AHA irrespective of its titer is an important risk factor for developing acute GVHD. Presence of AHA below MFI 2000 is probably not deleterious. Blood transfusion policy should be carefully evaluated before transplantation and therapeutic strategy to reduce the high titer of AHA before transplantation by plasma exchange could be implemented to reduce the risk of events due to the presence of preformed AHA and anti-MICA antibodies. However, due to the lack of sufficient power, the trends and the significant findings of this study should be validated in a larger cohort of children.
